# Smaller-loss planar SPP transmission line than conventional microstrip in microwave frequencies

**DOI:** 10.1038/srep23396

**Published:** 2016-03-17

**Authors:** Hao Chi Zhang, Qian Zhang, Jun Feng Liu, Wenxuan Tang, Yifeng Fan, Tie Jun Cui

**Affiliations:** 1 State Key Laboratory of Millimeter Waves, Southeast University, Nanjing 210096, China; 2 Synergetic Innovation Center of Wireless Communication Technology, Southeast University, Nanjing 210096, China; 3 Cooperative Innovation Centre of Terahertz Science, No.4, Section 2, North Jianshe Road, Chengdu 610054, China

## Abstract

Transmission line is a basic component in all passive devices, integrated circuits, and systems. Microstrip is the most popular transmission line in the microwave and millimeter-wave frequencies, and has been widely used in current electronic devices, circuits, and systems. One of the important issues to be solved in such applications is the relatively large transmission loss of microstrip. Here, we propose a method to reduce the loss of microwave transmission line based on the designable wavenumber of spoof surface plasmon polaritons (SPPs). Using this characteristic, we analyze and experimentally demonstrate the low-loss feature of the SPP transmission line through the perturbation method and S-parameter measurements, respectively. Both simulation and experimental results show that the SPP transmission line has much smaller transmission loss than traditional microstrip with the same size in the microwave frequencies. Hence, the spoof SPP transmission line may make a big step forward in the low-loss circuits and systems.

The loss of microwave transmission line (TL) is an important issue in the modern electronic systems, which may lead to signal distortions and heating problems^1^. For the traditional microwave TLs, such as microstrip (MS)^2^,^3^, however, it is difficult to control the loss through adjusting the structure parameters. Hence most methods to reduce the TL losses are focused on choosing low-loss materials^4^ or introducing extra air-layer as the substrate (e.g. the suspended microstrip^3^). However, in above techniques, the increased structure complexity and manufacture cost have limited their applications. Due to the feature of alterable wavenumbers^5^, spoof surface plasmon polaritons (SPPs) provide the possibility to solve the problem. In this article, we will demonstrate that a well-designed planar SPP TL has much smaller transmission loss than the traditional microstrip with the same size.

Generally speaking, the SPP mode is a special surface wave, which is formed by the interaction between free electrons and electromagnetic (EM) waves around metals in the optical frequencies^6^. Due to the negative-permittivity behavior of metals, this kind of surface wave can achieve some unique properties, such as the significant field enhancement and tightly field confinement^7^. Hence, the SPP-based optical circuits have been regarded as an important promising avenue for the further development^8^. At lower frequencies, however, the metal fundamentally shows the characteristics of a perfectly electric conductor (PEC), rather than the plasma with negative permittivity. Therefore, plasmonic metamaterials have been proposed to achieve the similar SPP properties at the lower frequencies using subwavelength artificial structures^5^,^9^,^10^,^11^. In this case, the working frequency of SPPs can be lowered to gigahertz by using surface decorations, such as periodic grooves^12^, holes^12^,^13^, slit^14^, blocks^15^ and heterostructures^16^,^17^,^18^. Those plasmonic metamaterials can support a structural version of SPPs, known as spoof SPPs^5^,^19^,^20^. Similar to bulk metamaterials^21^,^22^,^23^ and planar metasurfaces^17^, an importance feature of spoof SPPs is that their physical characteristics can be designed at will by tuning the geometrical parameters^24^,^25^. However, most existing plasmonic metamaterials cannot be regarded as a TL due to their complicated three-dimensional (3D) structures. To overcome this difficulty, a series of new type of planar SPP TLs have been proposed based on the printed circuit board (PCB) technology^26^,^27^,^28^,^29^, which can be considered as one of the most promising candidates for conformal circuits due to their flexibility and ultrathin thickness.

Here, we investigate the transmission loss of spoof SPP TL, and further structure a kind of low-loss SPP TL. For verification, we design and fabricate a low-loss SPP TL sample and a traditional microstrip TL under the same geometrical configuration. Both numerical simulations and experimental results show that the loss in the SPP TL is much lower than that in the microstrip in a wide frequency band from 2 to 10 GHz, which can help to build up low-loss circuits and systems in the future.

## Result

Different from the feature in optical frequencies, metals show the PEC characteristics in microwave frequencies of. Hence the metallic loss can be ignored in microwave TL because of the high electric conductivity, and the main loss is caused by the dielectric substrate. For potential applications in industry, in this study we select copper as the metal, and FR-4 as the substrate, which is one of the most popular substrates in the consumer electronics, with dielectric constant *ε*_*r*_ = 4.3, loss tangent tan δ = 0.025, and thickness *ts *= 0.5 mm.

**Figure 1:**
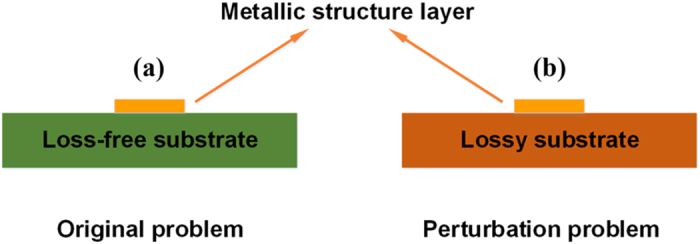
Schematic diagram for the original (**a**) and perturbation (**b**) problems. (**a**) The original problem in which the solution of electromagnetic fields is known. **(b)** The perturbation problem in which the loss of the substrate was introduced.

To investigate the loss problem in microwave TLs, we apply for the perturbation theory to calculate the loss coefficient. Here, we regard the loss of substrate as the perturbation quantity due to the relative ratio of the real and imaginary parts of the permittivity, as shown in Fig. 1. For a general microwave TL (e.g. microstrip and SPP TL), the variation of the wavenumber can be expressed approximately as


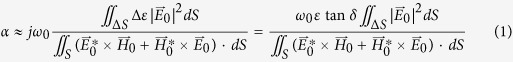


in which *α* is the attenuation constant of TL, Δ*S* and *S* are the regions of the substrate and total space, respectively, and *E*_0_ and *H*_0_ are the electric and magnetic fields of the original (lossless) problem. From Eq. (1), we observe that the denominator is twice of the average power flow of TL, which can be considered as an invariant in comparison. Hence we conclude that the loss of TL is caused by the field distribution in the substrate region. For two different types of TLs, lossless and lossy, the ratio of the attenuation constants can be calculated analytically by


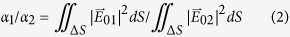


where *α*_*j*_ and *E*_0*j*_ are the attenuation constant and electric field of the original problem of the *j*th TL ( *j* = 1, 2). In Eq. (2), we observe that the radio of the attenuation constants is dependent on the electric field energy rather than the perturbation quantity. Hence, it is possible to control the transmission loss using the excellent feature of SPPs for the designable field confinement.

**Figure 2:**
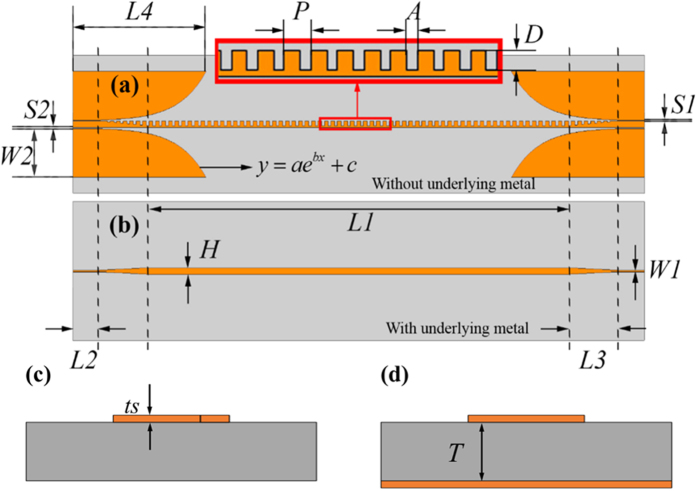
The configurations of two types TLs with the same overall dimensions. (**a**) The structure configuration of the spoof SPP TL, in which *S1* = 0.5 mm, *S2* = 0.7 mm, *L4* = 82 mm, and *W2* = 30 mm. The inset shows the Detailed structure of the spoof SPP TL, in which *P* = 4 mm, *A* = 1.6 mm and *D* = 3 mm. (**b**) The structure configuration of the conventional microstrip, in which *L1* = 320 mm, *L2* = 16 mm *L3* = 30 mm, *H* = 4 mm, and *W1* = 1 mm. (**c**) The cross section of the spoof SPP TL, in which *ts* = 0.018 mm. (**d**) The cross section of the conventional microstrip, in which *T* = 0.5 mm.

For convenience of industrial applications, we choose an ultrathin plasmonic waveguide^22^ as the SPP TL, which is composed of a periodically corrugated metallic strip. In a single unit, the groove depth and width are denoted as *D* and *A*, and the strip width and thickness are *H* and *T*, respectively, as illustrated in Fig. 2(a). Such a plasmonic waveguide is chosen due to the following reasons: 1) it has a strip-shaped outline similar to the microstrip, which can be easily used in the integrated circuits and systems; and 2) it allows the control of EM field distributions by changing the geometrical parameters.

**Figure 3:**
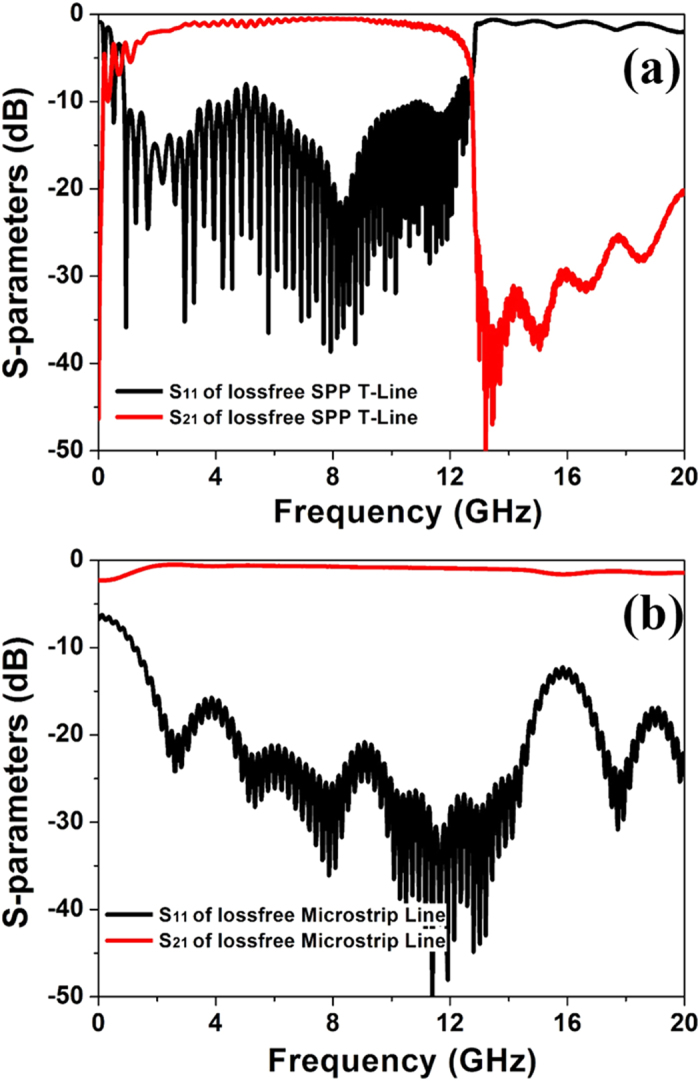
Simulated transmission and reflection coefficients of two types lossless TLs. (**a**) The SPP TL. (**b**) The microstrip.

Considering the compatibility to the microwave measurement system with 50- ohm impedance, a transition is designed between the 50-ohm coplanar waveguide (CPW) and spoof SPP TL. This transition achieves a smooth change by virtue of the gradient corrugations and flaring ground. A similar structure was demonstrated in ref. 30 for high-efficiency conversions between CPW and a symmetrical SPP structure. Here, we adapt an asymmetrical SPP structure for low-loss transmission, in which an asymmetric CPW is used to realize the high-efficiency conversion, as shown in Fig. 2(a,c). For comparison, we have also designed a microstrip line with the same length and strip width as the SPP TL, and a linear tapered conversion between the microstrip line and a standard 50-ohm microstrip, as shown in Fig. 2(b,d). When the substrate is lossless, the calculated transmission coefficients of the SPP TL and microstrip are illustrated in Fig. 3. In both TLs, the high transmission coefficients imply that the two conversions achieve a smooth bridge between different structures; and on the other hand, the radiation losses are very small.

**Figure 4:**
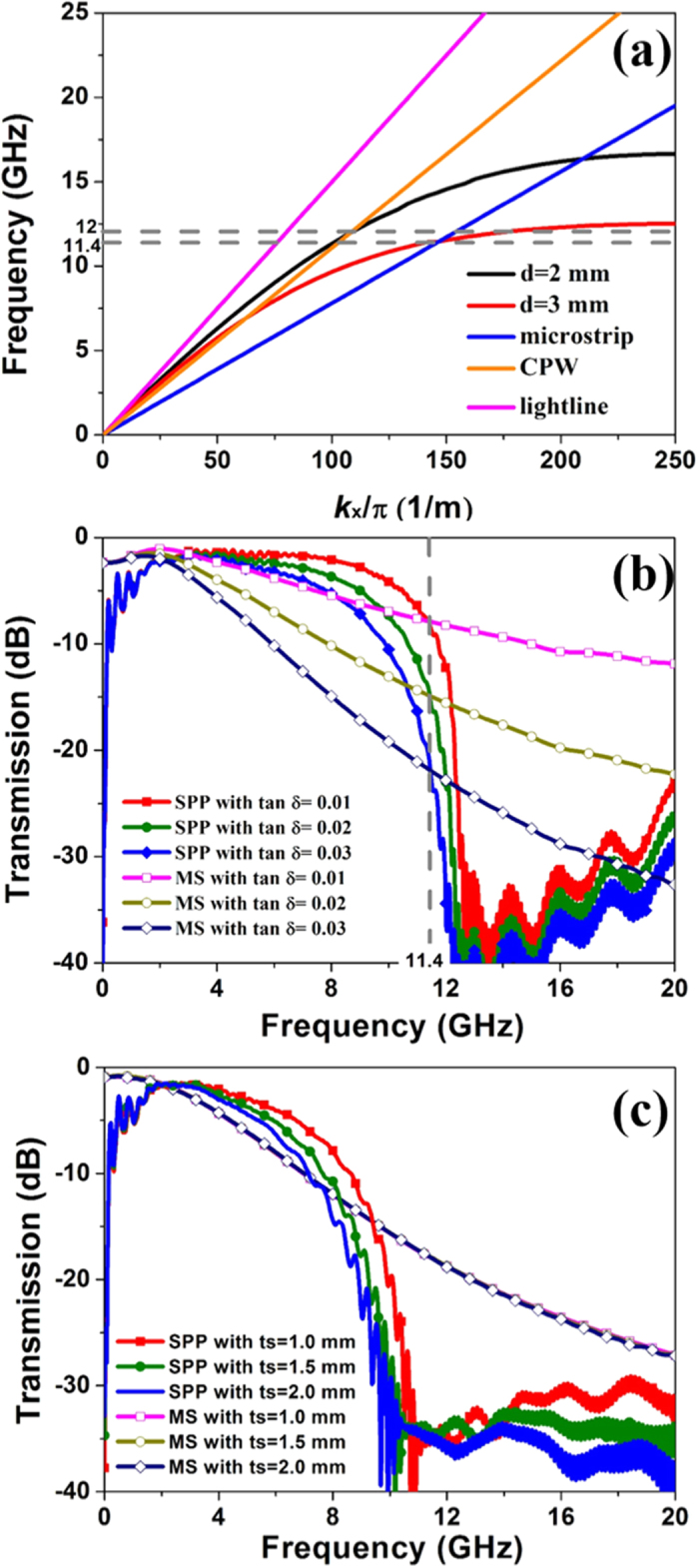
Simulated results of different TLs. (**a**) Dispersion diagrams of the spoof SPP structures for different groove depths and the microstrip with the same strip width. (**b**) Transmission spectra (S_21_) of two types TLs with different loss tangents. (**c**) Transmission spectra (S_21_) of two types TLs with different thickness of substrate.

For controlling the field confinement, we start from investigating wavenumbers of such two TLs. It is known that the decay constant along the tangential direction can be expressed as


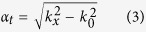


in which *k*_*x*_ is the wavenumber along the propagation direction, and *k*_0_ is free-space wavenumber, respectively. From Eq. (3), we observe that the capacity of the field confinement has a positive correlation to the wavenumber in the propagation direction. In order to simplify the estimation of field confinement, we consider the dispersion curves of the microstrip and SPP TLs with different groove depths *D*, which are obtained by the commercial software, CST Microwave Studio. In the design, the other geometrical parameters are chosen as *A *= 1.6 mm, *H *= 4 mm, *T *= 0.018 mm, and *P *= 4 mm. Though the ignored metallic loss leads to a very tiny blue shift of dispersion spectrum^31^ in the Eigen mode simulation, it provides us an approach for computing the wavenumbers of different TLs rapidly. In Fig. 4(a), we observe that the dispersion curves of spoof SPPs with different groove depths deviate gradually from the light line, like the natural SPPs at optical frequencies.

More importantly, the wavenumber of those SPP TLs are smaller than that of conventional microstrip within a special frequency range, implying that they support looser field mode propagations. Based on Eqs (2) and (3), we can estimate that the transmission loss of the spoof SPP TL may be smaller than that of the microstrip. For further verification, we have implemented numerical simulations of the transmission coefficients of SPP and microstrip TLs with different loss tangents, as presented in Fig. 4(b), in which the geometrical parameters are chosen the same as those in Fig. 2. From Fig. 4(b), we note that the transmission curves of such two kinds of TLs are all intersected at 11.4 GHz, regardless of the loss tangent in the dielectric substrate, as implied by Eq. (2). This intersection frequency is exactly the same as the theoretical prediction, 11.4 GHz. It is evident that, as the loss tangent increases from 0.01 to 0.03, the transmission property of SPP TL becomes worse slightly, while the transmission of microstrip deviates significantly. In the frequency range from 3 to 11.4 GHz, the transmission loss of SPP TL is much smaller than that of microstrip, especially when the dielectric loss is large and frequency is high.

**Figure 5:**
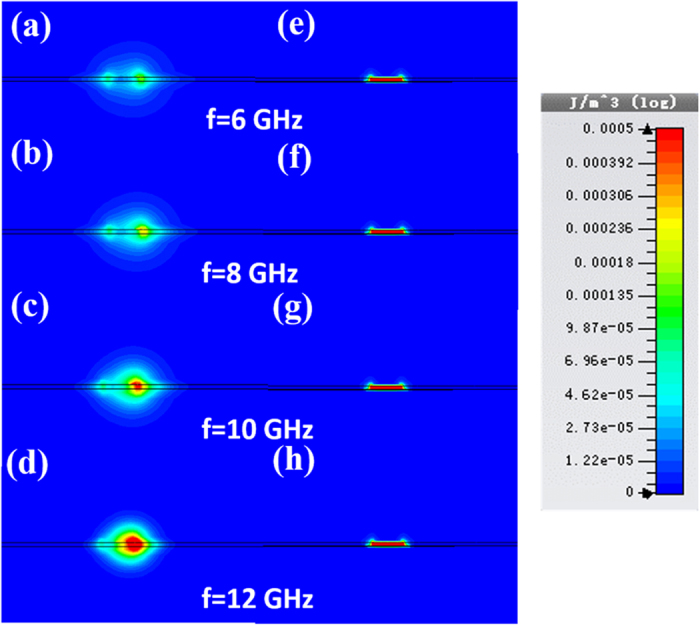
The magnitudes of electric energy densities on the cross sections of two types lossless TLs at different frequencies. (**a–d**) The distributions of electric energy densities of the SPP TL at 6 GHz (**a**), 8 GHz (**b**), 10 GHz (**c**), and 12 GHz (**d**). (**e–h**) The distributions of electric energy densities of the microstrip at 6 GHz (**e**), 8 GHz (**f**), 10 GHz (**g**), and 12 GHz (**h**).

Considering that different thicknesses of dielectric substrate are widely used in circuits and systems, we next investigate the influence brought by substrate thickness to the transmission performance. The structures simulated are the same as those in Fig. 2 and the loss tangent of dielectric substrate is 0.025. The transmission responses of SPP TL and microstrip are compared in Fig. 4(c). From this figure, the transmission loss (S21) remains stable in microstrip TL when the substrate thickness changes. In contrast, as the substrate thickness increases from 0.5 mm to 2 mm, the transmission loss (S21) in SPP TL becomes larger. In the lower frequencies (2 GHz–10 GHz), the field confinement in microstrip TL is much stronger than that in SPP TL. Hence in the microstrip, EM field is mostly confined in the dielectric substrate, which is also shown in Fig. 5. Based on Eq. (2), the transmission loss remains stable when the substrate thickness changes in microstrip. In SPP TL, however, the EM fields are distributed in both substrate and air areas at lower frequencies. Hence as the substrate gets thicker, the EM field stays more in substrate, and thus the transmission loss caused by substrate becomes bigger. Nevertheless, the transmission loss of SPP TL is still less than that of microstrip in the frequency range from 2 to 8 GHz. More importantly, due to the single-conductor feature, the SPP TLs are usually designed on thinner substrates.

For more general considerations, we demonstrate the distributions of the electric energy densities on different cross sections of the two lossless TLs in Fig. 5, in which the simulations are carried out by the time-domain solver of CST Microwave Studio. Here, the open boundary conditions are set to imitate the free-space environment. From Fig. 5(a–d), we notice that the field confinement ability in the SPP TL is gradually improved as the frequency increases, which is caused by the dispersion characteristic. However, the electric-energy-density distributions of the microstrip almost stay the same when the frequency changes, as shown in Fig. 5(e–h). On the other hand, we observe that the electric energy density of the SPP TL in the dielectric substrate is much less than that of microstrip from 6 to 11 GHz, which implies that the loss performance of the SPP TL is much better than that of microstrip, which is consistent with the numerical simulations depicted in Fig. 4(b). When the frequency goes higher, however, due to the gradually enhanced field confinement, the electric energy stays more in the substrate of SPP TL. As shown in Fig. 5d,h, the integration of electric energy density in the substrate region of SPP TL will be slightly bigger than that of microstrip, as predicted in the dispersion analysis in Fig. 4.

**Figure 6:**
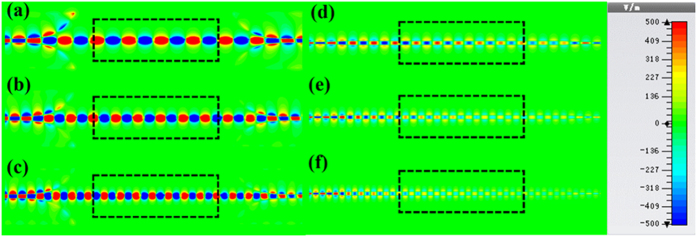
The simulated near-electric-field distributions on the x−y plane that is 1 mm above the structures at different frequencies, in which the black dotted outlines indicate the regions to be measured in experiments (see Fig. 8). (**a**–**c**) The SPP TL at 6 GHz (**a**), 8 GHz (**b**), and 10 GHz (**c**). (**d–f**) The microstrip at 6 GHz (**d**), 8 GHz (**e**), and 10 GHz (**f**).

To visualize the loss performance of such two TLs, we simulate the near-field distributions along the propagation direction, as demonstrated in Fig. 6. We clearly observe that the spoof SPP modes propagate very well through the measured region at all frequencies. For the conventional microstrip, however, the wave modes become weak obviously along the propagating path when the frequency increases from 6 to 10 GHz.

**Figure 7:**
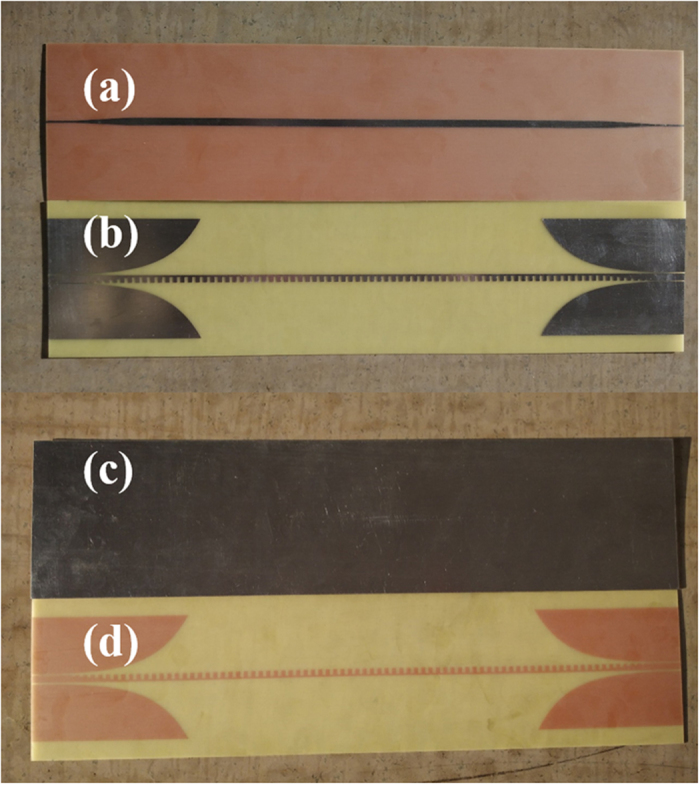
Photographs of the fabricated samples of the two types TLs. (**a–b**) The top views of the microstrip (**a**) and spoof SPP TL (**b**). (**c–d**) The bottom views of the microstrip (**c**) and spoof SPP TL (**d**). Here, the orange color is visually caused by the copper layer on the back, and the silver color is formed by tinned metallic surfaces.

**Figure 8:**
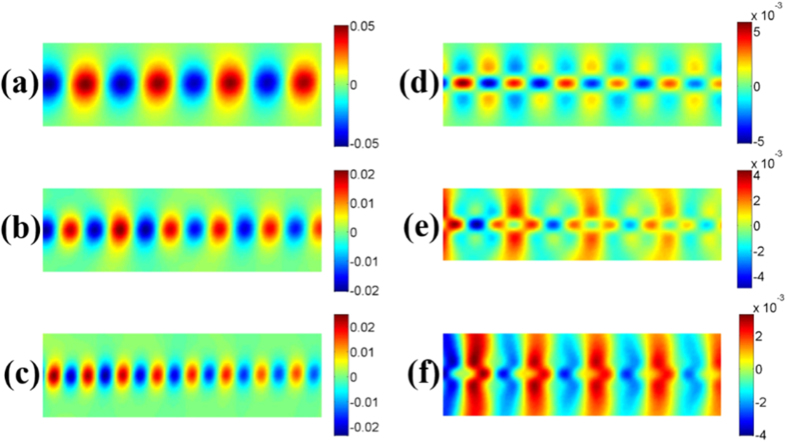
The measured near-electric-field distributions (*z* components) on an *x-y* plane that is 1 mm above the structures at different frequencies. Here, the measured regions are marked in Fig. 6. (**a**–**c**) The spoof SPP TL at 6 GHz (**a**), 8 GHz (**b**), and 10 GHz (**c**). (**d–f**) The microstrip at 6 GHz (**d**), 8 GHz (**e**), and 10 GHz (**f**).

To provide experimental verifications, we have fabricated two samples for SPP TL and microstrip with the same parameters as in above simulations, respectively. The bottom and top views of the samples are illustrated in Fig. 7. In order to connect with the Agilent vector network analyzer (VNA, N5230C), we weld two standard SMA connectors to the two ports of TLs for obtaining the transmission (S_21_) and reflection (S_11_) coefficients and the near-field mapping of TLs conveniently. In Fig. 8, we show the measured near electric fields on a plane that is 1 mm above the top surface of the TL structures. The measured region is indicated by the black dotted outline in Fig. 6. We clearly see that the measured results are in very good agreements with the simulation results, as indicated in Figs 6 and 8.

**Figure 9:**
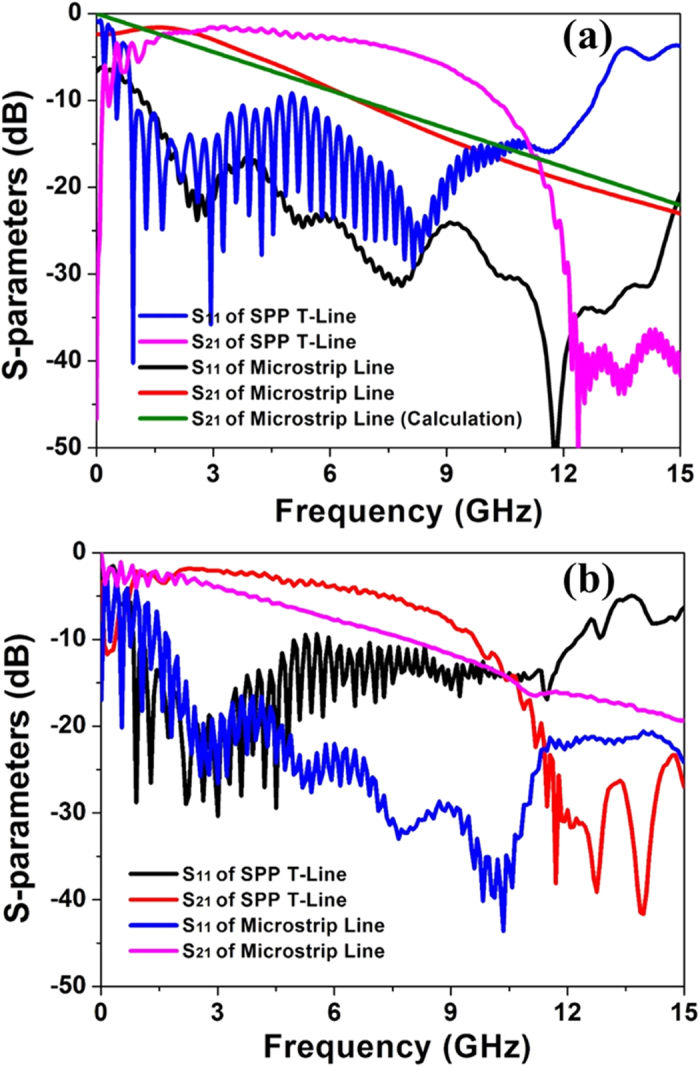
The simulated (**a**) and measured (**b**) transmission (S_21_) and reflection (S_11_) coefficients of two types TLs.

To study the transmission-loss performance of the two TL samples quantitatively, we demonstrate the simulation and measured results of the transmission and reflection coefficients in Fig. 9. For numerical simulation results shown in Fig. 9(a), we first compare the simulated transmission coefficient of the microstrip with the analytical formula which has been widely adopted in the microwave community


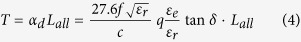


in which, *L*_*all*_ is the total length of the microstrip (including the transition parts for approxiamtion), *f* is the working frequency, *c* is the light speed, *ε*_*r*_ and tan *δ* are the dielectric constant and tangent loss of the substrate, and


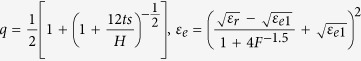


where


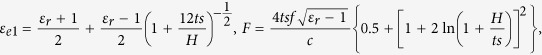


in which *H* is the width of microstrip, and *ts* is the substrate thickness. From Fig. 9(a), the simulated transmission coefficient of the microstrip has excellent match to the analytical prediction, confirming the accuracy of the simulation results. We also note from this figure that the transmission coefficient of SPP TL is about 4 dB higher than that of the microstrip from 3 to 10.5 GHz, indicating that the transmitted energy of the SPP TL is 2.5 times larger than that of microstrip. The measured transmission and reflection coefficients are presented in Fig. 9(b), which have good agreements with the simulated results. We observe that the pass bands of such two TLs in measured results have slight frequency shifts, which may be caused by the inhomogeneity of dielectric constant in the real FR-4 substrate.

**Figure 10:**
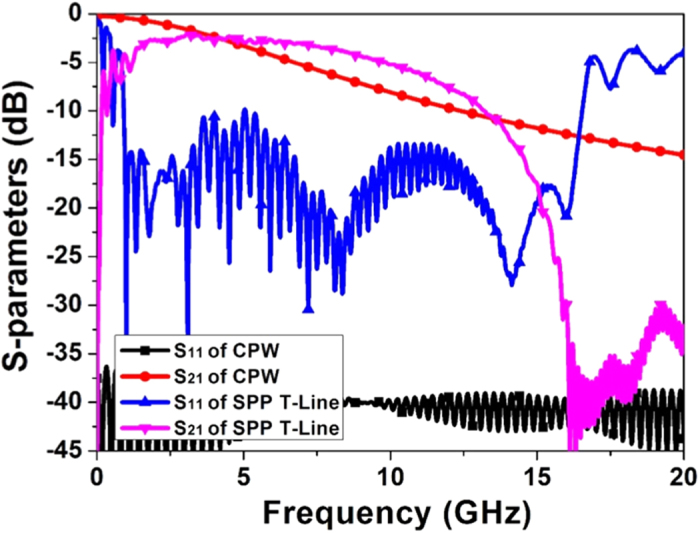
The simulated transmission (S21) and reflection (S11) coefficients of two types TLs.

At microwave frequencies, CPW is another kind of basic TLs in the circuits and systems, which is formed by a metallic strip and two separated coplanar grounds. Generally speaking, CPW has a smaller loss than the microstrip due to the smaller equivalent permittivity, as shown in Fig. 4(a). But by introducing the concept of SPP, it is possible to structure a smaller loss TL meanwhile keep the strip-shaped outline for the convenience of integrated circuits. For achieving a looser field distribution, we only need to decrease the groove depth *D*. For verification, a typical simulation result is shown in Fig. 10, in which the SPP TL structure parameters are the same as the fabricated sample shown in Fig. 7, except that the groove depth *D* changes to 2 mm. For comparison, the central conductor of CPW is equal to the strip width of SPP TL and the gap is set as 0.3 mm. From Fig. 10, it is clearly demonstrated that the transmission loss of SPP TL is much smaller than that of CPW in the frequency range from 4 to 13 GHz.

## Discussion

Through the analysis of perturbation method and the dispersion property, we have used the feature of designable dispersion spoof SPPs to achieve the low-loss TL at microwave frequencies. Both the simulation and experiment results demonstrate that the new-type TL can achieve much better transmission performance than the traditional microstrip with the same geometry. Owing to the excellent transmission performance without extra costs, the spoof SPP TL can be considered as one of the most potential candidates for microwave circuits, consumer electronics, and systems. In addition, the proposed method can be directly extended to higher frequencies (e.g., millimeter waves and sub-terahertz waves).

## Additional Information

**How to cite this article**: Zhang, H. C. *et al.* Smaller-loss planar SPP transmission line than conventional microstrip in microwave frequencies. *Sci. Rep.* **6**, 23396; doi: 10.1038/srep23396 (2016).
